# Supplementary material for: Hybrid Models and Biological Model Reduction with PyDSTool
Source: PLoS Comput Biol. 2012 Aug 9;8(8):e1002628. doi: 10.1371/journal.pcbi.1002628 (PMC3415397; doi:10.1371/journal.pcbi.1002628)
Supplement: Text S4 — Complete source code for the PyDSTool package (version 0.88.120504). Includes API documentation and help files linking to web pages. This file is identical to the current public release on Sourceforge.net. (ZIP) [file pcbi.1002628.s004.zip › PyDSTool/html/toc-PyDSTool.FuncSpec'-module.html]

xml version="1.0" encoding="ascii"?


FuncSpec'


# Module FuncSpec'

---

## Classes

ExpFuncSpec  
 FuncSpec  
 ImpFuncSpec  
 RHSfuncSpec  

## Functions

\_processReused

getSpecFromFile  
 makePartialJac  
 resolveClashingAuxFnPars  

## Variables

API

Continuous

Discrete

LargestInt32

NAMESEP

ONES

ZEROS

\_1DimplicitSolveMethods

\_all\_complex

\_all\_float

\_all\_int

\_all\_numpy\_complex

\_all\_numpy\_float

\_all\_numpy\_int

\_complex\_types

\_float\_types

\_implicitSolveMethods

\_indentstr

\_int\_types

\_num\_equivtype

\_num\_maxmin

\_num\_name2equivtypes

\_num\_name2type

\_num\_type2name

\_num\_types

\_pytypefromtype

\_real\_types

\_seq\_types

builtinFnSigInfo

builtin\_auxnames

name\_chars\_RE

null\_predicate

num\_chars

protected\_allnames

protected\_auxnamesDB

protected\_macronames

protected\_mathnames

protected\_randomnames

protected\_scipynames

protected\_specialfns

syms

targetLangs

---

[hide private]
